# Evaporation of a 1-Butanol Gel Fuel Droplet under Elevated Pressure Conditions

**DOI:** 10.1021/acsomega.1c06589

**Published:** 2022-03-04

**Authors:** Siwook Nam, Hyemin Kim

**Affiliations:** Department of Aeronautical Mechanical Design Engineering, Korea National University of Transportation, Daehak-ro 50, Chungju, Chungbuk 27469, Republic of Korea

## Abstract

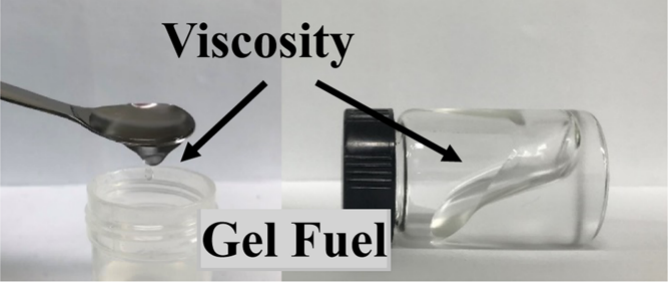

In this study, the evaporation characteristics of a gel fuel droplet under elevated temperatures and pressures were studied. 1-Butanol was selected as the base fuel because it is eco-friendly and has a calorific value similar to that of hydrocarbon-based fuels. In the experiment, 2.5 and 3 wt % hydroxypropyl methylcellulose (HPMC) was added as a gellant to generate the gel fuel. The viscosity of the gel fuel significantly increased compared to that of the 1-butanol fuel, and it decreased as the shear rate increased, which is referred to as the shear-thinning behavior. The evaporation of the 1-butanol gel fuel was divided into three periods, which were categorized as droplet heating, puffing, and crust formation. The behavior of each period changed as the ambient conditions changed. The elevation of the ambient temperature and gellant concentration boosted the intensity of puffing, whereas puffing was suppressed under higher-ambient-pressure conditions. When the ambient temperature increased from 600 to 700 °C, the evaporation rate of the gel fuels increased due to the increase in heat supply from the ambient temperature. Nevertheless, an increase in the ambient pressure and mass concentration of the gellant did not significantly affect the evaporation rate of the gel fuel.

## 1 Introduction

Conventional liquid and solid rocket systems have been successfully applied for several decades. Nevertheless, the advantages and disadvantages of each rocket system were definite, and the application of each system was quite limited. For example, it is not preferred to use the liquid rocket system in the military because of the long-term storage problem; additionally, solid rockets tend to not be used in the main stage of commercial rockets because it is difficult to control thrust during flight.^1−3^ In this circumstance, many researchers have agreed that a new type of fuel is essential to overcome the several shortcomings of conventional fuel rockets. Gel fuel, a mixture of liquid and gellant, is one of the candidates for the next-generation rocket propellants.^4,5^

The main feature of gel fuel is liquid fuel, which has a high viscosity. The structure of the gel is defined as “a substance that contains a continuous solid skeleton enclosing a continuous liquid phase,” in which the viscosity of the fuel is significantly increased compared to that of liquid fuels.^6^ Gel fuel is flowable like liquid fuel; thus, it is possible to control the thrust and fuel delivery via pipes. However, a gel fuel has a low vapor pressure at atmospheric pressure compared to liquid fuel, and long-term storage of rockets would be possible. The reduction of the leakage risk and stable dispersion characteristics when high-energy particles are mixed is another advantage of gel fuel.^7−9^ Because of this reason, gel fuel is expected to be an alternative to existing solid and liquid propellants, and several researchers have focused on the study of gel fuel in various ways.

Arnold and Anderson conducted experimental research on the rheological characteristics and combustion of silica gel fuel made by silica, which is a type of inorganic gellant. In this study, the gel fuel exhibited a shear-thinning phenomenon, which is typical for a non-Newtonian fluid. In the combustion process, it was found that an unburned rigid shell was formed during the combustion process of the droplets owing to the gellant. Moreover, the burning rate of the gel fuel decreased as the amount of gellant increased.^10^ In the research by Ghamari and Ratner, the authors evaluated the combustion characteristics of gel fuel droplets with polybutadiene (PBD). The produced fuels satisfy the d^2^-law in the initial section, but expansion and micro-explosion occurred with time because multicomponent fuels such as JP-8 and diesel were used as the base fuels. It was confirmed that the high viscosity of the gel fuel suppressed the evaporation of volatile species on the surface of the droplet and reduced the burning rate.^11^ Mishra et al. observed the changes in the burning rate and flame stand-off ratio of gel fuel. The gellant layer was formed as the gel fuel was heated owing to the difference in the boiling point at the surface of the droplet. Additionally, the calorific value and fuel vapor diffusion rate decreased with the addition of gellant owing to the decrease in the base fuel content. The burning rate and stand-off ratio of the flame of the gel fuel were lower than those of conventional fuels.^12^ Solomon and Natan studied the combustion characteristics of gel fuels containing metal particles. They found that the homogeneity inside the droplet could not be maintained, and phase separation occurred. Furthermore, only the gellant component with a high boiling point remained in the droplets during the combustion process, thus, the thickness of the gellant layer increased with time.^13^ Several researchers including Nachmoni et al.,^14^ Kunin et al.,^15^ Bar-or and Natan^16^ have also conducted studies on the combustion characteristics of gel fuels.

Previous research has shown the potential of gel fuel as a next-generation rocket propellant in spite of several drawbacks such as poor atomization and combustion characteristics. Intensive observation and analyses of gel fuel behavior in various combustion environments have to be conducted. However, previous gel fuel studies have mostly focused on atmospheric pressure conditions, and the results cannot reflect the characteristics of gel fuel high-pressure combustors such as rocket engines. Moreover, most of the previous research focused on hydrocarbon base gel fuel; therefore, research on alternative gel fuels such as alcohol base fuel is highly needed.

The main goal of this study was to identify the evaporation characteristics of a single 1-butanol gel fuel droplet under various temperature and pressure conditions. 1-butanol has a high caloric value compared to other alcohol-type fuels; the quality of gel fuel is superior to that of other hydrocarbon-type gel fuels. Moreover, 1-butanol gel fuel is an excellent candidate for the gel propellant in terms of sustainability because it can be produced by the fermentation process. In this study, 1-butanol gel fuel was studied from the production to the experiment to understand the evaporation behavior of gel fuel under high-pressure conditions.

## 2 Experimental Setup

**Figure 1:**
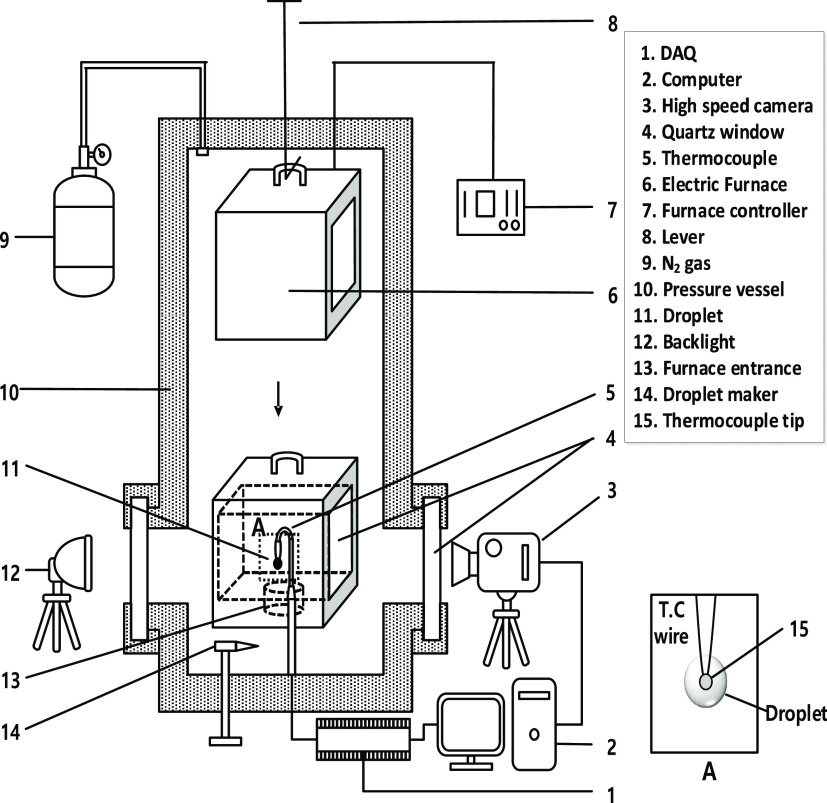
Schematic of the experimental setup.

Figure 1 shows a schematic diagram of the experimental apparatus composed of a high-pressure test chamber, an optical observation facility, and measuring sensors. The present experimental setup was identical to that used in previous studies,^17,18^ but a minor change in the gel fuel experiment was performed.

### 2.1 Test Chamber

The test chamber consisted of an electric furnace, a pressure vessel, and droplet temperature measuring equipment. As shown in Figure 1, an electric furnace was installed inside the pressure vessel, and a high-temperature environment around the test droplet was created by dropping the furnace to the droplet installation section. The temperature of the electric furnace was regulated by the PID controller and had an error within ±5 °C of the set temperature. A K-type thermocouple with a diameter of 70 μm (Omega Engineering, Inc.) was used for the installation of the droplet and to measure the droplet temperature during the evaporation process of a single droplet.

It should be noted that the measured droplet temperature of the gel fuel was not highly reliable compared to that of liquid fuel because the gellant shell around the droplet hindered the film adhesion of the droplet at the thermocouple tip. Furthermore, droplet vibration due to the micro-explosion process during the experiment disturbed the adequate measurement of the droplet temperature. Nevertheless, the results were still valid for comparing and analyzing the results between the different experimental conditions.

The installed diameter of the gel droplet was set to 1000 ± 100 μm. The inner chamber was filled with pure nitrogen gas to prevent the ignition of the fuel droplet, and the pressure was adjusted using the regulator. The experiment was repeated at least five times under the same conditions to minimize the experimental deviation. Detailed information of the test chamber was presented in a previous study conducted by the authors.^18^

### 2.2 Preparation of Gel Fuel

In the present study, 1-butanol was used as the base fuel, and hydroxypropyl methylcellulose (HPMC) was added as a gellant. 1-butanol is an eco-friendly and sustainable fuel that is produced by fermentation, and the heating value reaches ∼80% compared to hydrocarbon fuels.^19^ Additionally, the quality of 1-butanol gel fuel is outstanding over the hydrocarbon base gel fuel, which has the potential to be a candidate for the gel propellant. HPMC is an organic gellant that can burn during the combustion process.

To prepare the 1-butanol/HPMC gel fuel, 10 wt % distilled water was added to 1-butanol. The water in 1-butanol generates a hydrogen bond between 1-butanol and HPMC, which makes the fuel thicker. Subsequently, HPMC was added to the 1-butanol/water mixture using an impeller. It was important to pour the HPMC into the liquid very slowly to prevent aggregation of the gellant. The produced gel fuel was stored for ∼24 h to remove air bubbles generated during the mixing process. The available mixing range of HPMC was quite narrow for producing proper gel fuel. When the concentration of gellant was less than 2.5%, the viscosity of the fuel drastically reduced that the fuel did not sustain a gel form. On the other hand, the fuel was so thick that its flowability was diminished when the concentration of HPMC exceeded 3%. Thus, the concentrations of HPMC for the experiments were set to 2.5 and 3%. The surface tension of the butanol/water aqueous solution showed a constant or slight increase according to the temperature, unlike the pure liquid, which decreased as the temperature increased.^20^ It was helpful to calculate the boiling temperature change of 1-butanol under ambient pressure.^21^ For this, the following equation was used under 1, 5, 10, and 15 bar conditions, and the results are listed in Table 1.
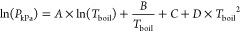
The coefficients for *n*-butanol are *A* = −9.882614, *B* = −9127.49639, *C* = 86.72214, and *D* = 1.42848 × 10^–6^

**Table 1:** Boiling Temperature of *n*-Butanol under Various Pressure Conditions

	*n*-butanol
1 bar	118 °C
5 bar	171 °C
10 bar	201 °C
15 bar	222 °C

**Figure 2:**
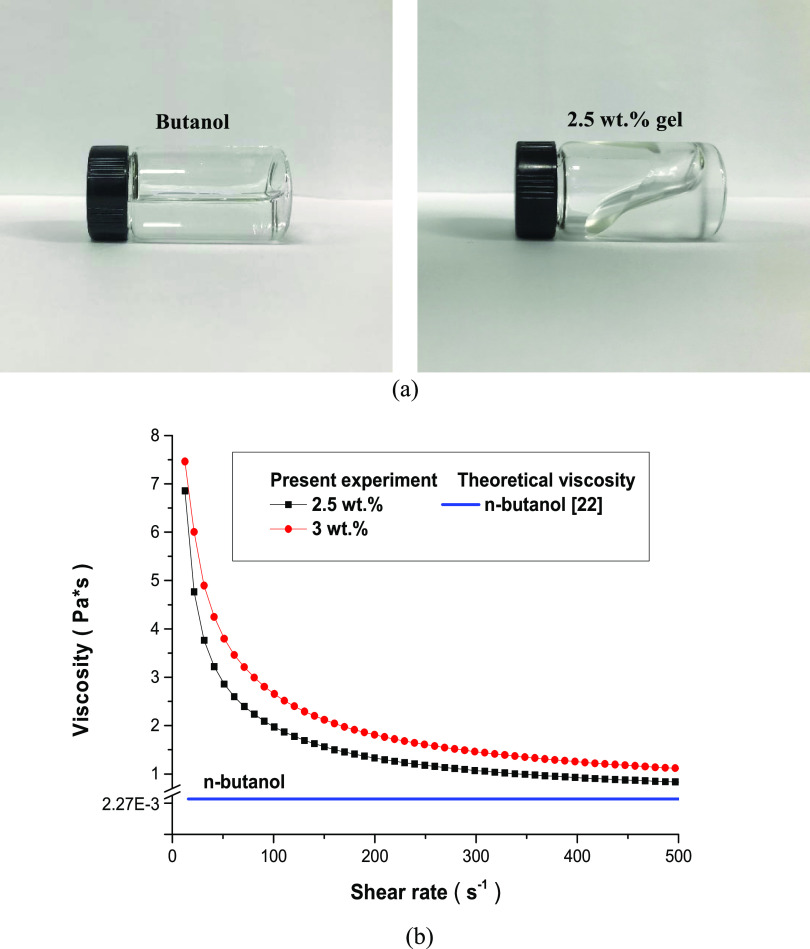
(a) Images of 2.5 wt % gel and butanol/water mixture. (b) Viscosity-shear rate of the gel fuels.

As shown in Figure 2a, the flowability of 2.5 wt % butanol gel fuel drastically decreased compared to pure butanol because of the increase of viscosity due to the gallant addition. The rheological characteristics of the pure butanol and 1-butanol/HPMC gel fuel at room temperature (25 °C) were quantitatively measured using a rotational rheometer (Model: HAAKE MARS-III). It is known that pure 1-butanol exhibits Newtonian fluid behavior with a constant viscosity value of 2.274 mPa·s.^22^

However, the gel fuel exhibited the characteristics of a non-Newtonian fluid, as shown in Figure 2b, whose viscosity changed according to the shear rate. The viscosity of the gel fuel was significantly increased compared to that of pure butanol, and it showed a shear-thinning phenomenon in which the viscosity decreased as the shear rate increased. This is because the hydrogen bond of the gel fuel produced high viscosity when it was maintained, but it was broken when a certain amount of shear force was applied and the viscosity regressed to ∼0.8 Pa·s.^23^ The viscosity when the amount of gellant was 3 wt % was higher than that of 2.5 wt %, but the shear-thinning behavior was similar in all of the shear rate ranges.

### 2.3 Optical Measuring Methods

A high-speed camera (Model: FASTCAM mini UX 100) was used to obtain temporal images of the droplet. The acquisition rate of the droplet image was 200 fps, and it was converted into droplet diameter data using a post-processing program. The program is a modified version used in previous research, and it deduced the droplet diameter change as well as the evaporation rate.^18^

**Figure 3:**
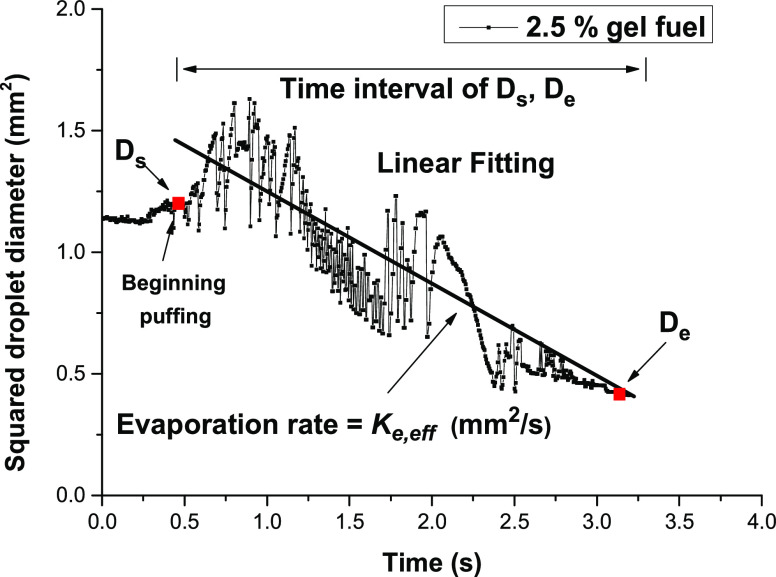
Determination of *D*_s_, *D*_e_, and *t* for measuring effective evaporation rate.

It is important to measure the proper evaporation rate with a definite standard because it will provide quantitative information to other researchers. However, inflation and puffing during the experiments distorted the results that the determination of the evaporation rate was quite difficult. In this study, an effective evaporation rate was introduced to correct the distorted droplet diameter during the experiment. The effective evaporation rate is defined as^24^
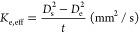
where *D*_s_ is the droplet diameter at the start of the measurement, *D*_e_ is the droplet diameter at the end of the measurement, and *t* is the time between *D*_s_ and *D*_e_. The point of *D*_s_ was selected where the diameter of the droplet was first changed by puffing and micro-explosion, and *D*_e_ was determined when the squared droplet diameter dropped to 0.4 mm^2^, as shown in Figure 3. The effective evaporation rate was deduced by linearly fitting the droplet diameter regression between the points *D*_s_ and *D*_e_. Despite the uncertainties of this process in measuring the evaporation rate, it would be an efficient method to quantitatively present the evaporation characteristics of the gel fuel. Errors in the evaporation rate were considerably reduced as the ambient pressure increased.

## 3 Results and Discussion

Gellant contents of 2.5 and 3 wt % were selected as the experimental fuel condition, and the ambient temperature and pressure conditions were 600 and 700 °C and 1, 5, 10, and 15 bar, respectively. The results in various experimental conditions are addressed in this chapter.

### 3.1 General Evaporation Behavior of Gel Fuel Droplet

**Figure 4:**
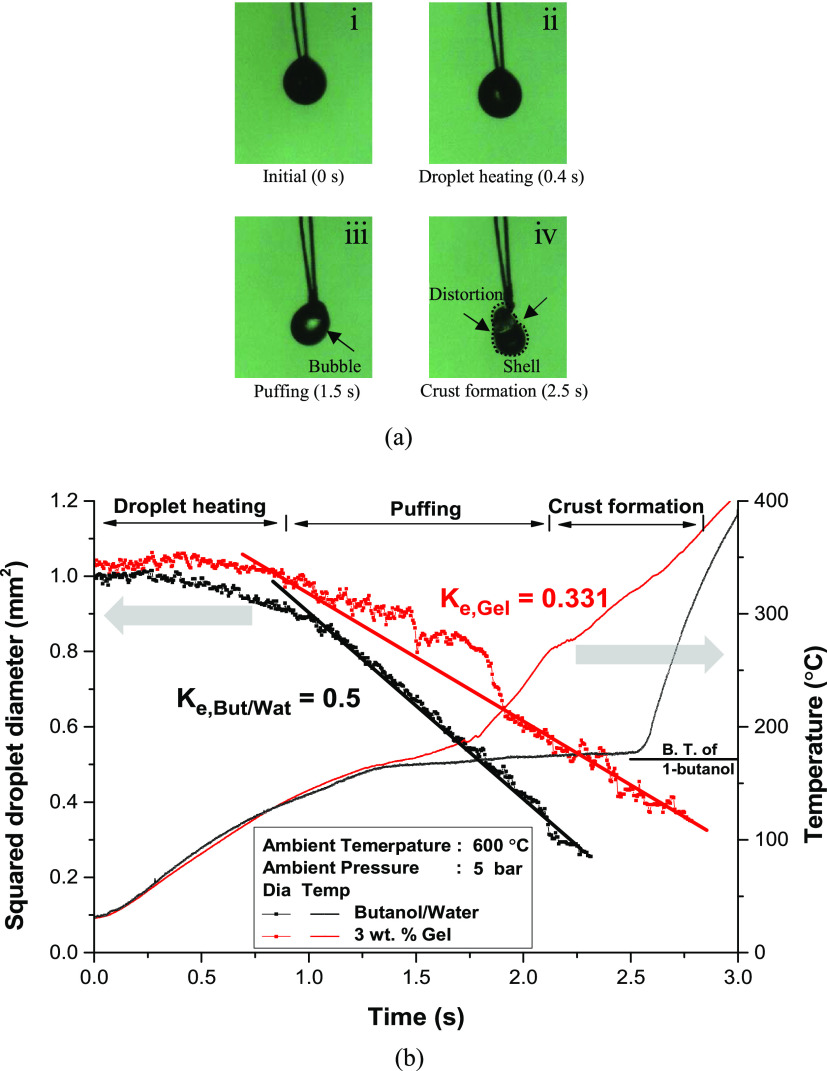
(a) Images of 3 wt % gel fuel droplet at 5 bar and 600 °C. (b) Squared droplet diameter and temperature of 1-butanol/water mixture and gel fuel droplet.

The evaporation process of the gel propellant varied significantly depending on the experimental conditions in which the general behavior of the gel droplet evaporation would be quite difficult to explain. Nevertheless, the evaporation process of the 1-butanol gel propellant can be categorized into several periods. Figure 4a shows the droplet images in each period, and Figure 4b presents the temporal variation of the 2.5 wt % gel droplet diameter and temperature when the ambient temperature and pressure were 600 °C and 5 bar, respectively. Furthermore, the optical images of the droplets in a specific period are presented. The droplets generally undergo three periods: droplet heating, puffing, and crust formation. Each period is marked in the graph with the droplet images at a specific time. Furthermore, changes in the diameter and temperature of the 1-butanol/water mixture droplet were added to the graph to compare the evaporation characteristics.

The gel droplet first underwent a droplet heating period with various liquid fuels. The droplet temperature rapidly increased when the ambient temperature was changed from 20 to 600 °C. A slight increase in the droplet diameter was observed during this period owing to thermal expansion. The temperature profile between the gel and 1-butanol/water mixture droplets did not exhibit any differences during the droplet heating period. In a study by Mandal and Bakshi,^25^ the viscosity of the fuel affected the droplet regression rate under convection conditions. However, it seemed that the difference in viscosity between the 1-butanol/water and gel droplet did not influence the droplet heating rate because the forced flow may not have been generated in the electric furnace, and the internal circulation flow in the droplet was not actively induced. In this circumstance, conduction rather than convection dominated the droplet heating, and the difference in viscosity for each fuel could be neglected. However, the regression of the droplet diameter during the droplet heating period was slightly faster for 1-butanol/water droplets because the gel structure at the droplet surface hindered the evaporation of 1-butanol. Intensive puffing did not occur during this period.

After the droplet heating period, 1-butanol/water droplets exhibited constant regression of the droplet diameter, which is a conventional characteristic of a pure component droplet (d^2^ law).^26^ Additionally, the droplet temperature was maintained near the boiling temperature of 1-butanol. The puffing did not observe since the boiling temperature of water and 1-butanol was similar. However, the puffing period began at the surface of the gel fuel droplet ∼0.8 s after the beginning of the experiment. Puffing was mainly observed during this period, as shown in the graphs and images. The mechanism of puffing can be explained based on previous research. Fuel vapor was generated inside the droplet as the droplet temperature reached the boiling temperature of 1-butanol. However, the fuel vapor could not escape the droplet because of the gel structure at the droplet surface; hence, it accumulated inside the droplet.^13^ The inflation of the droplet diameter presented in the graph supports this explanation. When a sufficient amount of fuel vapor is generated inside the droplet, the surface tension of the gel structure cannot withstand the pressure of the vapor, and an explosion occurs.

The behavior of the puffing process is mainly affected by the experimental conditions, which cannot be generalized easily. Nevertheless, puffing was mainly observed in the early stages of the puffing period, and the phenomenon changed to the repetition of the droplet inflation and explosion with time. This is because the gel structure around the droplet surface thickened as the 1-butanol evaporated, the fuel vapor could not penetrate the droplet surface, and the droplet inflated like a balloon. The droplet temperature during the puffing period increased further, whereas the temperature of the 1-butanol/water droplet was maintained near the boiling point of 1-butanol. It seemed that the temperature of the fuel vapor captured in the droplet was measured rather than the gel fuel itself during the puffing period, thus, the droplet temperature during this period was not reliable.

The crust formation period followed the puffing period. The puffing and rapid change of the droplet diameter were mitigated during this period owing to the thick gellant crust at the droplet surface. A large portion of 1-butanol in the droplet evaporated, and the gellant crust was generated at the droplet surface. This crust suppressed puffing by a strong surface tensile force. Furthermore, the shape of the droplet was not circular, as shown in the image because the surface tension of the fuel droplet did not maintain the droplet shape; however, the gel crust generated a distorted shape of the droplet. The temperature of the droplet rose during this period because 1-butanol gradually ran out in the droplet. The duration of the crust formation period relied on the experimental conditions, and it is discussed in the following sections.

### 3.2 Effect of Ambient Temperature

**Figure 5:**
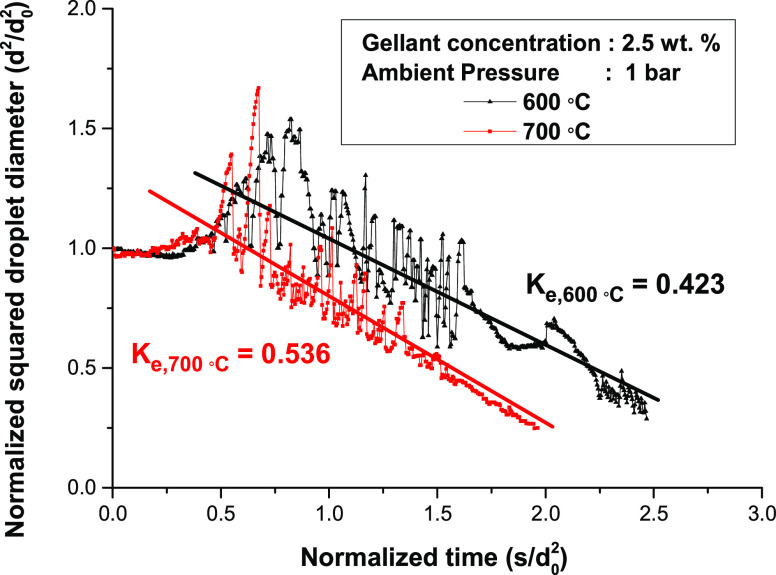
Temporal change of normalized droplet diameter for different ambient temperature conditions.

The effect of the ambient temperature on the gel droplet evaporation behavior is discussed in this section. Figure 5 presents the droplet diameter change at ambient temperatures of 600 and 700 °C, while the other conditions were identical. It has to be noted that droplet temperature data was not presented because the droplet motion during the droplet inflation and puffing distorted the measured droplet temperature. In both cases, the diameter of the droplet inflated during the droplet heating period. However, the duration was shorter for the 700 °C case because of the difference of the heat feedback from the ambient conditions. The inflation of the droplet owing to the fuel vapor was observed at the end of the droplet heating period.

Intensive puffing occurred at 700 °C as soon as the puffing period started because the ambient temperature was relatively high and the droplet temperature reached the boiling point of 1-butanol. In this circumstance, the fuel vapor inside the droplet sufficiently accumulated, and it could easily penetrate the gel structure at the droplet surface. Compared to the 600 °C case, the puffing was mitigated early because the amount of fuel evaporated early, as presented in the droplet diameter graph. However, the droplet inflated for ∼0.3 normalized time for the case of 600 °C. When considering the measured droplet temperature data, it seems that the droplet temperature in the early period of the puffing period did not reach the boiling point of 1-butanol, which delayed the generation of fuel vapor. Sufficient vapor pressure for puffing did not accumulate under these conditions, and the inflation of droplets rather than puffing was mainly presented.

The crust formation period started at 1.1 s/mm^2^ for the 700 °C case and at ∼1.6 s/mm^2^ for the case of 600 °C. In both cases, the gel structure at the surface was thick and puffing was significantly reduced; even small puffing was occasionally observed. The inflation of droplets during the crust formation period observed at 600 °C was due to the formation of fuel vapor inside the droplet. The droplet diameter in this period shrank quite linearly like the pure substance droplets, but the mechanism of evaporation in this period would be different because the shape of the droplet was quietly distorted owing to the gel crust.

The effective evaporation rates for 600 and 700 °C are shown in Figure 5. Although the results of the evaporation rates were not accurate owing to the rapid change of the droplet diameter and distortion of the droplet shape, it is sufficient to show that an increase in the ambient temperature boosted the evaporation of droplets, similar to the case of conventional liquid fuel. A more detailed discussion of the droplet evaporation rate under different ambient temperature conditions is presented in Section 3.4.

### 3.3 Effect of Ambient Pressure

**Figure 6:**
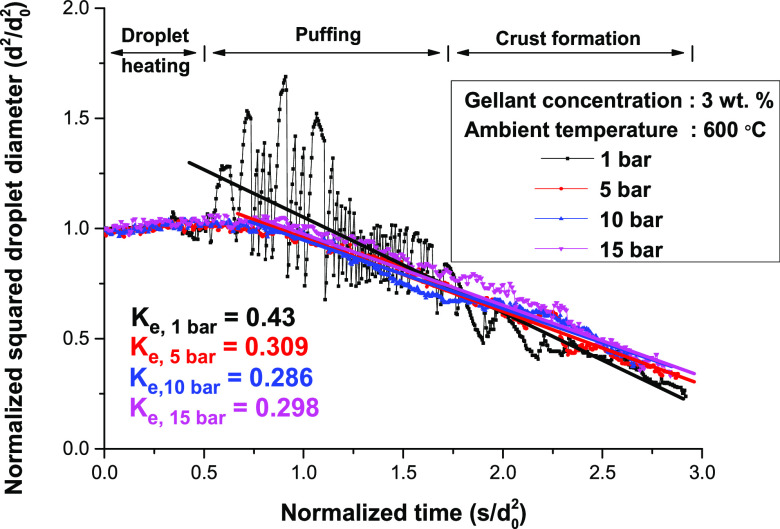
Temporal change of normalized droplet diameter for different ambient pressure conditions.

**Figure 7:**
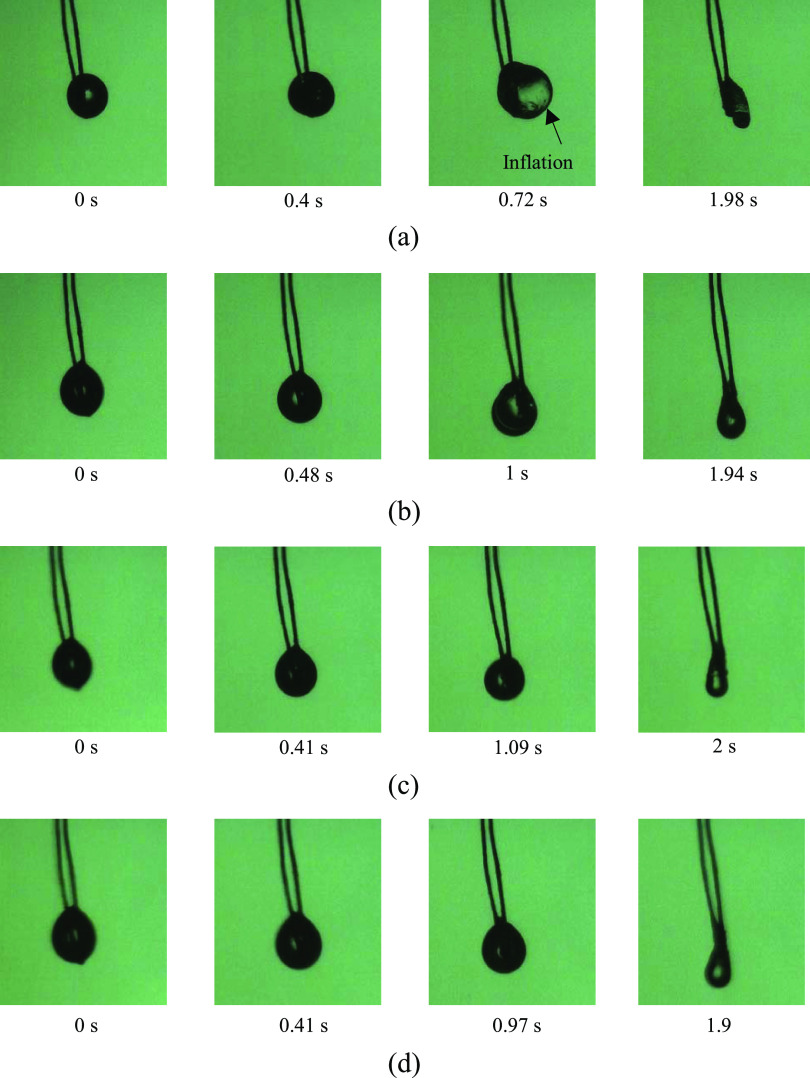
Images of droplet under different ambient pressure conditions: (a) 1 bar, (b) 5 bar, (c) 10 bar, and (d) 15 bar.

The droplet diameter and temperature change under different ambient pressures are presented in Figure 6, and images of the droplet during the puffing period are shown in Figure 7. The mass concentration of the gellant was 3 wt % and the ambient temperature was 600 °C. There was no distinctive difference in the droplet diameter change for different ambient pressures during the droplet heating period; however, the droplet temperature increased faster under high-ambient pressure conditions because of the increase in the boiling temperature of 1-butanol. An increase in the boiling temperature induced a longer droplet heating period until the droplet temperature converged to the boiling point of 1-butanol.^27^

The puffing intensity during the puffing period was considerably different from the ambient pressure changes. As shown in Figure 6, intensive puffing, including droplet inflation, was observed for the 1 bar case. The puffing diminished with the ambient pressure, and only small inflation of the droplet was shown in the graph for the case of 15 bar. The main reason for this is that the ambient pressure suppressed puffing, as already discussed in previous research conducted by the authors.^17^ A high ambient pressure decreased the vapor pressure at a specific temperature, hindering the generation of vapor inside the droplet. Moreover, the inward force at the droplet surface by the ambient pressure prevented the rupture of the droplet surface owing to the inner vapor pressure, and eventually lowered the puffing process. The puffing behavior of gel fuel at elevated pressure had been unknown since most of the previous studies about the evaporation and combustion of gel fuel droplet concentrated on atmospheric conditions. In this study, however, we showed that the puffing of the gel fuel under high-pressure condition was similar to liquid fuels.

There were obvious differences between the puffing and crust formation periods for the 1 bar case because the puffing was suddenly mitigated as the crust formation period started. However, the separation of the puffing and crust formation period was uncertain for the higher-pressure case because intensive puffing like the 1 bar condition was suppressed by the ambient pressure. The inflation of the droplet diameter was observed intermittently, as shown in the 15 bar graph in Figure 6, owing to the confined vapor inside the thick crust.

The evaporation rate marked in the graph indicates that it was slightly higher for the 1 bar case, but it was similar for other pressure conditions. This is a distinct evaporation characteristic of gel fuel compared to conventional liquid fuel. The evaporation rate of conventional liquid fuel usually increases with the ambient pressure because the latent heat of the fuel decreases as the ambient pressure increases.

Previous researchers experimentally discovered that ambient pressure decreased the latent heat of liquid fuels.^28^ However, the present experiment showed a similar evaporation rate regardless of the ambient pressure, although the latent heat of fuel decreased. This may be addressed by intensive theoretical research, which is beyond the scope of the present study, but it can be estimated that the gel structure around the droplet surface may affect the evaporation characteristics of gel fuel. A more detailed discussion of the overall experimental results of the evaporation result is presented in Section 3.4.

### 3.4 Effect of Gellant Weight Concentration

**Figure 8:**
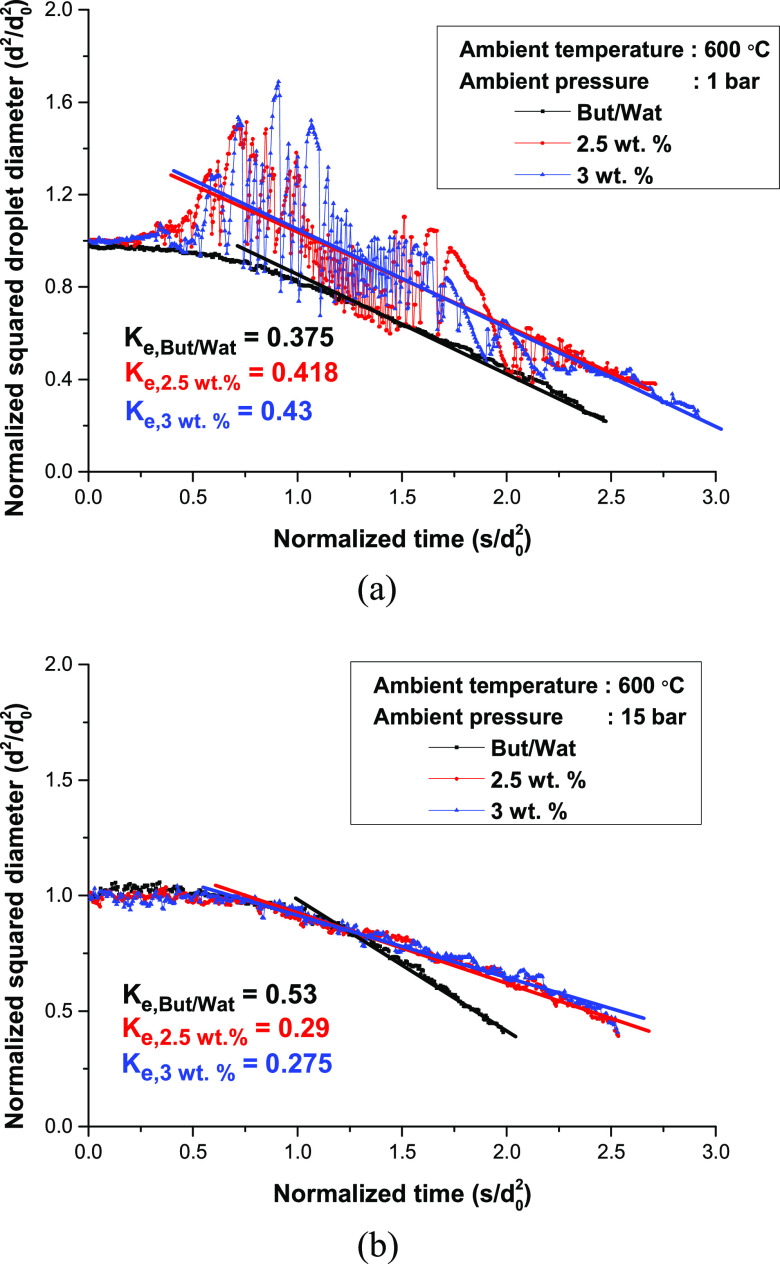
Temporal change of normalized droplet diameter for gellant ratios of 0, 2.5, and 3 wt % in (a) 1 bar (b) 15 bar.

The addition of gellant significantly changed the evaporation behavior of the droplet, and the phenomenon was altered under ambient conditions. Figure 8 shows the temporal changes in water/1-butanol, 2.5, and 3 wt % gel droplet diameters in 1 and 15 bar conditions. For the 1 bar case, the water/1-butanol diameter experienced droplet heating and d^2^ regression of droplet diameter followed after similar with conventional liquid fuel. Puffing did not occur during the experiment because the boiling points of 1-butanol and water were similar. The evaporation rate was measured to be 0.375 mm^2^/s.

In the case of the 2.5 and 3 wt % cases, however, an intensive puffing process during the experiment was observed as presented in Figure 8a. In both cases, the droplet underwent a short droplet heating period, and massive inflation of the droplet diameter occurred after. The droplet diameter during the early puffing period was larger for the 3 wt % case because the gel structure at the droplet surface was strong, but the difference was not distinctive. Active puffing was generated after droplet inflation in both cases, and the droplet proceeded to the crust formation period. Mitigation of the puffing and distortion of the droplet shape due to the crust formation was mainly observed in both cases. The effective evaporation rates for the 2.5 and 3 wt % cases were 0.418 and 0.43 mm^2^/s, respectively.

Comparing the effective evaporation rates, they were similar in the three cases, but the underlying mechanism was completely different. Unlike the 1-butanol/water droplet, the evaporation hindered the gel fuel droplet owing to the gel structure at the droplet surface. Nevertheless, intensive puffing ejected part of the fuel, induced a faster reduction in the droplet diameter, and boosted the overall droplet evaporation rate. This is called atomization, owing to puffing.^17^ Thus, it can be concluded that the puffing process of gel fuel at low ambient pressure increases the atomization of fuel as well as the effective evaporation rate. This is an advantageous factor for efficient combustion because the surface-to-volume ratio of the fuel droplet is elevated. Furthermore, the evaporation rate during the crust formation period decreased owing to the thick gel structure at the droplet surface.

The phenomenon completely changed when the ambient pressure was changed to 15 bar, as shown in Figure 8b. In the case of the 1-butanol/water mixture droplet, no distinctive differences were observed even under high-pressure conditions, except for an increase in the evaporation rate from 0.375 to 0.53 mm^2^/s. The reduction of the latent heat under high-pressure conditions elevated the evaporation rate, as discussed previously. However, several differences compared to the 1 bar condition were observed for the gel fuel droplet cases. First, puffing was significantly diminished because of the high ambient pressure. It can be observed that the puffing periods were uncertain, as shown in Figure 8b, owing to the reduction in puffing. Additionally, the effective evaporation rate decreased from 0.42 to 0.28 mm^2^/s because a decrease in the intensive puffing process deteriorates the atomization process of the droplet. Moreover, the strong gel structure at the surface may act as a blocker of fuel vapor evaporation when considering that the evaporation rate for gel fuel compared to the 1-butanol/water case was worse.^29^

The results clearly showed that the gel structure at the surface boosted the evaporation rate under lower-ambient-pressure conditions by enhancing the droplet atomization process, such as puffing and droplet inflation; however, it acted as a suppressor of evaporation under higher-pressure conditions by interrupting the fuel evaporation at the droplet surface. This finding reflects that precise consideration of the ambient pressure for designing the gel propellant combustor has to be done because the evaporation characteristics of gel fuel are highly affected.

**Figure 9:**
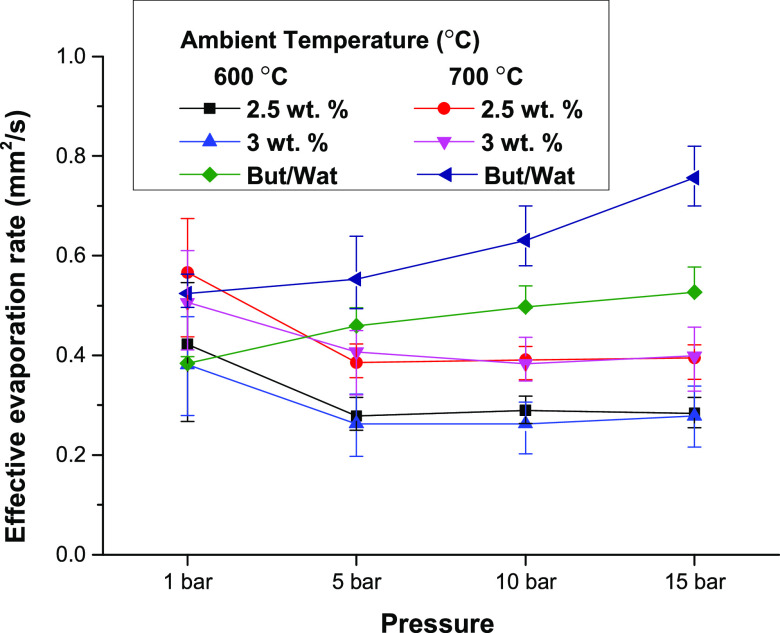
Effective evaporation rate under various experimental conditions.

Figure 9 summarizes the effective evaporation rate for all experimental conditions. In the 1 bar condition, the evaporation rates for all fuel cases were similar, and the mechanism of evaporation between the 1-butanol/water and gel fuel droplets was quite different. It should be noted that the deviation of the evaporation rate for the gel fuel droplet was relatively large because of the difficulty of measurement. The evaporation rate further increased as the ambient pressure increased for the 1-butanol/water droplets, and this phenomenon was similar at 600 and 700 °C. In the case of the gel fuel droplets, in contrast, evaporation diminished when the ambient pressure increased to 5 bar and was maintained regardless of the ambient pressure changes. This result indicates that the puffing process is an important factor for enhancing the evaporation of gel fuel droplets. Additionally, it seems that the gel structure at the droplet surface takes charge of the evaporation process of the gel fuel at elevated pressure, by observing the fact that the evaporation rate was independent of the ambient pressure even though latent heat was decreased under high-pressure conditions. The effective evaporation rates for gellant concentrations of 2.5 and 3 wt % were similar for all experimental cases. The results clearly showed that temperature was the only factor that boosted the overall evaporation rate of the gel fuel.

The experiment was conducted under lower-temperature condition than that of the combustion because of the limitation of the heating device. Nevertheless, evaporation characteristics of the gel fuel droplet would also be applicable to combustion cases when considering the several previous studies that discussed the compatibility of evaporation and combustion of a single droplet.^30^

## 4 Conclusions

In this study, the evaporation characteristics of 1-butanol gel fuel droplets were experimentally evaluated under various temperature and pressure conditions. The results are summarized below.

(1) The evaporation process of the gel fuel droplet consists of three periods: droplet heating, puffing, and crust formation, and the characteristics of each period varied with ambient conditions.
(2) Increasing the ambient temperature elevated the evaporation rate, but the ambient pressure did not significantly affect the evaporation rate because of the compensation effect of the latent heat change and puffing process. The difference in the gellant wt % also exhibited minor effects on the gel fuel evaporation.
(3) The overall evaporation rate for the gel fuel was insensitive to ambient conditions compared to the 1-butanol/water mixture. However, the evaporation rate of the gel fuel rose at 1 bar owing to the massive puffing process during evaporation.
